# Ultra-Endurance Associated With Moderate Exercise in Rats Induces Cerebellar Oxidative Stress and Impairs Reactive GFAP Isoform Profile

**DOI:** 10.3389/fnmol.2020.00157

**Published:** 2020-09-02

**Authors:** Raphael Fabricio de Souza, Ricielle Lopes Augusto, Silvia Regina Arruda de Moraes, Fabio Borges de Souza, Lílian Vanessa da Penha Gonçalves, Danielle Dutra Pereira, Gisele Machado Magalhães Moreno, Fernanda Maria Araujo de Souza, Belmira Lara da Silveira Andrade-da-Costa

**Affiliations:** ^1^Laboratory of Neurophysiology, Department of Physiology and Pharmacology, Center of Biosciences, Federal University of Pernambuco, Recife, Brazil; ^2^Postgraduate Program in Neuropsychiatry and Behavioral Sciences, Federal University of Pernambuco, Recife, Brazil; ^3^Department of Physical Education, Federal University of Sergipe, São Cristovão, Brazil; ^4^Group of Studies and Research of Performance, Sport, Health and Paralympic Sports – GEPEPS, Federal University of Sergipe, São Cristovão, Brazil; ^5^Laboratory of Neuromuscular Plasticity, Department of Anatomy, Center of Biosciences, Federal University of Pernambuco, Recife, Brazil; ^6^Laboratory of Neuropharmacology and Integrative Physiology, Center of Biosciences, Federal University of Alagoas, Maceió, Brazil

**Keywords:** oxidative stress, central nervous system, high-volume training, catalase, superoxide dismutase, lipid peroxidation, glial fibrillary acidic protein

## Abstract

Ultra-endurance (UE) race has been associated with brain metabolic changes, but it is still unknown which regions are vulnerable. This study investigated whether high-volume training in rodents, even under moderate intensity, can induce cerebellar oxidative and inflammatory status. Forty-five adult rats were divided into six groups according to a training period, followed or not by an exhaustion test (ET) that simulated UE: control (C), control + ET (C-ET), moderate-volume (MV) training and MV-ET, high-volume training (HV) and HV-ET. The training period was 30 (MV) and 90 (HV) min/day, 5 times/week for 3 months as a continuous running on a treadmill at a maximum velocity of 12 m/min. After 24 h, the ET was performed at 50% maximum velocities up to the animals refused to run, and then serum lactate levels were evaluated. Serum and cerebellar homogenates were obtained 24 h after ET. Serum creatine kinase (CK), lactate dehydrogenase (LDH), and corticosterone levels were assessed. Lipid peroxidation (LP), nitric oxide (NO), Interleukin 1β (IL-1β), and GFAP proteins, reduced and oxidized glutathione (GSH and GSSG) levels, superoxide dismutase (SOD) and catalase (CAT) activities were quantified in the cerebellum. Serum lactate concentrations were lower in MV-ET (∼20%) and HV-ET (∼40%) compared to the C-ET group. CK and corticosterone levels were increased more than ∼ twofold by HV training compared to control. ET increased CK levels in MV-ET vs. MV group (*P* = 0.026). HV induced higher LP levels (∼40%), but an additive effect of ET was only seen in the MV-ET group (*P* = 0.02). SOD activity was higher in all trained groups vs. C and C-ET (*P* < 0.05). CAT activity, however, was intensified only in the MV group (*P* < 0.02). The 50 kDa GFAP levels were enhanced in C-ET and MV-ET vs. respective controls, while 42 kDa (∼40%) and 39 kDa (∼26%) isoform levels were reduced. In the HV-ET group, the 50 KDa isoform amount was reduced ∼40–60% compared to the other groups and the 39 KDa isoform, increased sevenfold. LDH levels, GSH/GSSG ratio, and NO production were not modified. ET elevated IL-1β levels in the CT and MV groups. Data shows that cerebellar resilience to oxidative damage may be maintained under moderate-volume training, but it is reduced by UE running. High-volume training *per se* provoked systemic metabolic changes, cerebellar lipid peroxidation, and unbalanced enzymatic antioxidant resource. UE after high-volume training modified the GFAP isoform profile suggesting impaired astrocyte reactivity in the cerebellum.

## Introduction

In the last decades, a noticeable increase in the number of athletes taking part in long-distance running and ultramarathons has been reported (Agnew et al., 2018). In contrast to regular volume training, some deleterious effects have been described as a result of high volume, repetitive physical effort, and exhaustive and complex competitions (Knechtle and Nikolaidis, 2018). Recent studies have identified physiological impairment resulting from the ultra-endurance race (UE), encompassing cardiac and major artery structural remodeling, indications of pathological condition (O’Keefe et al., 2012; Bonsignore et al., 2017), marked renal and muscle damage (Jastrzȩbski et al., 2015), hepatic dysfunction (Rama et al., 2015), cartilage wear (Franciozi et al., 2013), and oxidative damage to the peripheral and central nervous systems (CNS) (Chalimoniuk et al., 2015a; Muñoz et al., 2017).

The CNS is especially vulnerable to oxidative damage due to its high O_2_-dependent mitochondrial activity, intensified by aerobic exercise, increased oxygen uptake, and cerebral blood flow (40–70%) to meet energy demands (Chalimoniuk et al., 2015a). When subjected to an antioxidant enzymatic imbalance, the CNS is an important target for reactive oxygen species (ROS) and oxidative stress secondary byproducts. Physical exercise can exert neuroprotective or harmful actions in the redox balance, depending on the intensity, volume, duration, and specificity of the brain region, independent of the involvement of this region in the locomotor function (de Souza et al., 2018).

The cerebellum plays a relevant role in motor, cognitive, and limbic activity (Hibi and Shimizu, 2011; Schmahmann, 2019) and promotes movement control, motor learning (Celnik, 2015) balance, muscle coordination, posture (Hibi and Shimizu, 2011; Powell et al., 2015; Jirenhed et al., 2017) and visually guided locomotion (Armstrong, 1996; Rabe et al., 2008; Schmahmann, 2019). Moderate aerobic exercise increased the dendritic density, field area, and total branch length of Purkinje cells (Pysh and Weiss, 1979; Huang et al., 2018) and favors cerebellar angiogenesis (Black et al., 1990; Lee, 2007). Also, cerebellar molecular changes induced by exercise promote plasticity in the motor cortex (Mang et al., 2016), attenuate cerebellar deficiencies (Tercero-Pérez et al., 2019), suppress Purkinje cell loss during Parkinson’s disease (Lee et al., 2018), increase mitochondrial biogenesis (Marques-Aleixo et al., 2015), and inhibit oxidative stress, promoting apoptotic signaling (Marques-Aleixo et al., 2016). Purkinje cells have also been shown to have high levels of monocarboxylate transporters (MCTs), which are key proteins in energy metabolism when stimulated, by treadmill running (Hoshino et al., 2016).

Studies that evaluated training over 60 min per day have examined the effects on the entire rodent brain (Radak et al., 2001; Hagen et al., 2018), neglecting metabolic specificities of different brain regions. Notwithstanding the importance of the cerebellum for cognitive planning and execution functions involved in movement control, limited experimental studies have investigated the oxidative impact of high-volume training and UE tests in this brain region. Another aspect that motivated our study was its differential vulnerability and neurochemical profile (Scorziello et al., 2001). To our knowledge, no study has yet investigated whether UE could modify astrocyte function in the cerebellum. Bergman glia is a specialized radial astrocyte of the cerebellum and has a differential genesis, ensheaths, and controls almost all synapses on the Purkinje neurons, conferring to this region distinct plastic responses when compared to the cerebral cortex, for example (Bernardinelli et al., 2014). Moreover, functional modifications in the Bergman glia can affect associative motor learning and/or motor performance (De Zeeuw and Hoogland, 2015). Astrocytes are active players in brain energy delivery, production, utilization, and storage (Sonnay et al., 2017). Brain glycogen is chiefly located in these glial cells and it decreases with extensive and prolonged exercise (Matsui et al., 2012, 2017). However, it is still unknown whether UE running induces altered glial activity as well as modifies antioxidant defense mechanisms when associated with high-volume running training (Jastrzȩbski et al., 2015; Rama et al., 2015; Knechtle and Nikolaidis, 2018; Millet et al., 2018).

The current discussion on the negative effects of high-volume and UE on runner health (Knechtle and Nikolaidis, 2018; Millet et al., 2018), and the scarcity of knowledge about these effects in locomotion-related brain regions has motivated the present study. We hypothesized that rodent cerebellar resilience to oxidative injuries may be reduced after high-volume training, regardless of the exhaustion test simulating UE running.

## Materials and Methods

### Experimental Animal Groups

Forty-five adult male Wistar rats (60 days; 225.84 ± 24.33 g) were obtained from the animal production unit of the Federal University of Paraíba, João Pessoa, Brazil. The animals were kept on a 12 h light/dark cycle under an inverse photoperiod (lights on 6:00 p.m.), under a controlled environment with temperature (22°C ± 2°C) and had free access to food and water.

After the running training test, the animals were randomized into six groups according to the moderate-intensity training period, followed or not by the exhaustion test (ET): control (C: *n* = 8, placed on the treadmill off), control + ET (C + ET: *n* = 7), moderate-volume training (MV: *n* = 8) moderate-volume training + ET (MV + ET: *n* = 7), high-volume training (HV: *n* = 8), and high-volume training + ET (HV + ET: *n* = 7). All the experimental procedures animals followed the guidelines for the Care and Use of Laboratory Animals previously approved by the Animal Use Ethics Committee of the Federal University of Pernambuco, Brazil (# 0035-2017).

### Physical Training Protocol

For running training, a motorized treadmill (AVS products^®^) with individual tracks and equipped with rear shock (2.0 mA) was used. One week before the beginning of the training, the animals were adapted to a speed of 5 m/min for 10 min, 5 times per week. Subsequently, the animals were submitted to a maximum velocity test (Vmax) to determine the training threshold. Vmax consisted of a graded threshold run and speed increments (5 m/min every 3 min), starting at 5 m/min up to the maximum intensity achieved by each animal (de Senna et al., 2011).

The treadmill trainability test was rated on a scale of 1 to 5 according to the following criteria: (1) refusing to run, (2) below-average runner (sporadically running but stops running constantly), (3) average runner (maintains a steady run but falls or stops running sporadically), (4) above-average runner (consistent runner, occasionally falls backward on the treadmill), and (5) good runner (Dishman et al., 1988). Animals classified as score ≥3 were randomized in the groups for physical training. Only two animals scored <3, and these animals were put in the control groups.

The training protocol was continuous for 12 weeks. Daily exercise sessions 3 to 5 times/week with low to moderate intensity (50–70% of maximum intensity achieved in Vmax tests) were performed by the MV, MV-ET, HV, and HV-ET groups (see Table 1). The training times of the MV and MV-ET or HV and HV-ET groups were gradually increased from 10 to 30 min or 10 to 90 min per day, respectively, over 12 weeks (Tarini et al., 2013). Each training session was preceded by 5 min of warm-up at 30% of Vmax and the end of the run 5 min of recovery at 30% of Vmax (de Senna et al., 2011). These times were included in the total protocol period. The rear shock was used only in the early steps of animal adaptation to the treadmill, 1 week before the beginning of the training despite the score obtained in the trainability test. It was not necessary to use rear shock for the 3 months training period.

**TABLE 1:** Training protocol.

Week		Training threshold (%– m/min)	Time (Min)		Frequency of weekly sessions	Recovery training (hours)
			MV	HV		
Adaptation	1°	5 m/min	10 min	10 min	3×	24
	2°	50%	10 min	10 min	3×	24
1°Phase	3°	50%	15 min	20 min	3×	24
	4°	50%	20 min	30 min	3×	24
	5°	50%	25 min	40 min	5×	24
	6°	50%	30 min	50 min	5×	24
2°Phase	7°	60%	30 min	60 min	5×	24
	8°	60%	30 min	70 min	5×	24
	9°	60%	30 min	80 min	5×	24
	10°	70%	30 min	90 min	5×	24
3°Phase	11°	70%	30 min	90 min	5×	24
	12°	70%	30 min	90 min	5×	24

*MV, moderate-volume training; HV, high-volume training.*

**FIGURE 1:**
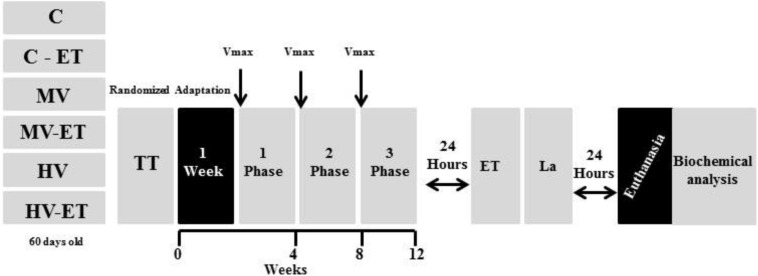
Experimental protocol of aerobic exercise training under different volumes. Vmáx: Max speed test, ET, exhaustion test; TT, trainability test; La, lactate analysis. C, control group; C-ET, control group + exhaustion test; MV, moderate-volume group; MV-ET, moderate-volume + exhaustion test group; HV, high-volume group; HV-ET, high-volume + exhaustion test group.

Twenty-four hours after the training period, the C-ET, MV-ET, and HV-ET groups were submitted to the exhaustion test. This test was performed up to the maximum running distance (low to moderate intensities) when the animals were considered depleted and refused to run (touching the running box 10 times for 1 min) (Tarini et al., 2013), after which tail blood was collected for analysis of serum lactate levels. After 24 h, all animals were weighed, anesthetized with 100% isoflurane, followed by blood collection directly from the heart (left ventricle) before decapitation for immediate removal of the cerebellum tissue (see Figure 1).

### Running Performance

For analysis of running performance, the running time (min), distance traveled (meters), speed (meters/min), and horizontal workload were evaluated in the groups submitted to the exhaustion test.

### Markers of Metabolic Changes in the Serum

Total creatine kinase (CK) and lactate dehydrogenase (LDH) concentrations in the serum were quantified as indicators of potential damage to the tissues. The blood samples (∼4 mL) were collected after euthanasia by cardiac puncture and put in dry tubes for separating serum (InjeXvácuo) with a clot activator. They were subsequently centrifuged for 10 min at 800 *g* and 4°C, and then the serum was separated for analysis using specific commercial kits (Katal Biotechnology, São Paulo, Brazil for LDH and Labtest Diagnóstica S.A.; Minas Gerais, Brazil for CK) according to the manufacturer’s instructions.

For quantification of lactate levels, the tail vein blood sample collection was used; approximately 25 μl of the blood sample was placed directly onto test strips (BM-Lactate) and immediately analyzed with an Accutrend Lactate Accu-Check lactate meter (Roche, Brazil) just after ET.

For the corticosterone assay, 50 μl of serum was used. Analyses were made in duplicate per sample at a 1:1000 dilution. The entire procedure was performed as recommended by the Corticosterone ELISA kit (Cayman Chemical, No 501320).

### Biochemical Analysis of Cerebellum Oxidative Status

#### Cerebellum Tissue Preparation

The cerebellum dissection was made over a filter paper that was put over an iced buffer, and the wet weight was obtained before the immersion of cerebellum in the saline solution and kept in a deep freezer at –80°C. Then, this tissue was homogenized at 4°C in a lysis buffer containing 10 mM ethylenediaminetetraacetic acid (EDTA), 2 mM phenylmethylsulfonyl fluoride (PMSF), 100 mM sodium fluoride, 10 mM sodium pyrophosphate (Na_4_P_2_O_7_), 100 mM Tris (hydroxymethyl) aminomethane, pH 7.4, and a 1% cocktail of protease inhibitors (AEBSF- [4- (2-Aminoethyl) benzenesulfonyl fluoride hydrochloride; Aprotinin, Bestatin hydrochloride, E-64-[N-(*trans-*Epoxysuccinyl)-L-leucine) 4-guanidino butylamine, leupeptin hemisulfate pepstatin A; (Sigma-Aldrich, United States) (1:5 w/v). Thereafter the homogenate was centrifuged for 10 min at 10,000 *g* at 4°C and the supernatants stored at −80°C for further analyses.

#### Lipid Peroxidation Analysis

Lipoperoxidation (LP) was measured by estimating the malondialdehyde (MDA) levels using the thiobarbituric acid reactive substance (TBARS) assay, which measures the MDA present in the sample, as well as MDA generated from lipid hydroperoxides by the hydrolytic conditions of the reaction (Ohkawa et al., 1979). The reaction was developed by sequential addition of 80 μL 8.1% sodium dodecyl sulfate, 600 μL 20% acetic acid, pH 3.5, and 600 μL 0.8% TBA solutions and 200 μL of cerebellum supernatant, boiled in water for 60 min and then cooled in an ice bath. Afterward, 600 μL of n-butanol was added, the mixture was shaken and centrifuged at 2,500 *g* for 10 min, and the upper phase was collected and analyzed at 532 nm using a plate reader (Thermo Scientific, Varioskan flash spectral scanning multimode reader).

#### Nitric Oxide Levels

Nitrite levels were estimated using Griess reagent as an indicator of nitric oxide (NO) production (Green et al., 1982). Equal volumes (100 μL) of supernatant and Griess reagent were placed in a 96-well plate and reacted for 10 min at room temperature (∼ 22°C). The absorbance of the diazonium compound was measured at a wavelength of 540 nm. Results were expressed as nmol per mg protein regarding a standard curve constructed with known concentrations of sodium nitrite.

#### Total Superoxide Dismutase and Catalase Activity

Evaluation of total superoxide dismutase (t-SOD) activity was performed according to Misra and Fridovich (1972) at 25°C. Triplicates of cerebellum supernatants (60 μL) were previously incubated in a water bath at 37°C. Sodium carbonate (920 μL of 0.05% buffer) was added. The reaction was developed by adding 20 μl of 150 mM epinephrine in 0.05% acetic acid. The decay kinetics of absorbance levels at 480 nm was evaluated by measurements every 15 s over a total period of 2 min. One unit of t-SOD was defined as the amount of enzyme that causes 50% inhibition of epinephrine oxidation. The enzymatic activity of t-SOD tissue was expressed in units per milligram of protein (U/mg protein).

Catalase enzymatic activity (CAT) was measured according to Aebi (1984). Triplicates of cerebellum supernatants (60 μL) were added to 905 μL sodium phosphate buffer pH 7.0. The reaction was developed by adding 35 μl of 300 mM hydrogen peroxide in sodium phosphate buffer. The rate constant K decomposition of H_2_O_2_ under our experimental conditions of temperature (22°C), and pH (7.0) was determined to be 2.3 to measure absorbance changes for 10 s for 1.5 min at 240 nm. Enzyme activity was also expressed as units per milligram of protein (U/mg protein).

#### Glutathione Levels

Glutathione (GSH) levels were analyzed according to Hissin and Hilf (1976). Thus, 450 μL of 100 mM phosphate buffer with EDTA (5 mM) (pH 8.0) was added to 50 μL of the supernatant; 50 μL of this mixture plus 140 μL of 100 mM phosphate buffer plus 10 μL of o-phthaldialdehyde (OPA; Sigma Aldrich, United States) solutions were placed in plates and incubated for 20 min at room temperature, protected from light. Absorbance was recorded on a spectrofluorometer using a wavelength of 350 nm. Results were expressed in μmol per mg protein regarding a curve pattern constructed with known concentrations of GSH.

#### Oxidized Glutathione Levels

Oxidized Glutathione (GSSG) levels were analyzed according to Hissin and Hilf (1976). Thus, 50 μL of the supernatant was incubated at room temperature with 20 μL of 0.04M N-ethylmaleimide (NEM) for 30 min to interact with GSH present in the tissue. To this mixture, 430 mL of 0.1 M sodium hydroxide (NaOH) was added; 50 μL of this mixture plus 140 μL of 0.1 M NaOH and 10 mL OPA were placed in the wells of a 96-well plate. This mixture was incubated for 15 min at room temperature and protected from light. The reading was taken on a spectrofluorometer using a wavelength of 350 nm for emission. Results were expressed in μmol per mg protein regarding a standard curve constructed with known concentrations of GSSG.

### GFAP and IL-1β Protein Levels in the Cerebellum

Glial fibrillary acidic protein (GFAP) and IL-1 protein levels in the cerebellar tissue were analyzed by Western blotting. Cerebellar homogenate samples were diluted in sample buffer and boiled for 5 min. Forty micrograms of protein per lane were electrophoretically separated in 15% sodium dodecyl sulfate polyacrylamide gel containing 10% sodium dodecyl sulfate, 30% Acrylamide/Bis-Acrylamide Solution, 1.5 M Tris–HCl (pH 8.8), 10% APS, and TEMED at 100V, 0.15A, and 300W. After separation, the proteins were transferred to a nitrocellulose membrane (Maine Manufacturing, United States, 0.22 micron) for 1.5 h at 250V, 0.35A, and 300W. The membranes were blocked for 1 h in a Tris-Tween 20 (TBS-T) solution containing 5% BSA. They were then incubated with rabbit polyclonal anti-GFAP primary antibody (Dako, Agilent Technologies, Germany, 1:1000) or rabbit polyclonal anti-IL-1β (Sino Biological Inc., Beijing, China, 1:2000), both diluted in TBS-T overnight at 4°C. After three washes in TBS-T, the membrane was treated with goat anti-rabbit secondary antibody conjugated to Horseradish peroxidase (Jackson ImmunoResearch, United States; 1:50,000), diluted in TBS-T for 1 h. Subsequently, the membranes were washed in TBS-T and stained with Luminata Western HRP substrate (Millipore, United States) **via** enhanced chemiluminescence using a ChemiDoc imaging system (Bio-Rad, United States). Mouse monoclonal anti-beta-actin antibody (Santa Cruz Biotechnology, CA 1:10,000) was used to normalize the quantitative values. The data were analyzed using the Image Lab 6.0.1 software (Bio-Rad, United States).

### Statistical Analysis

The Shapiro-Wilk test was used to verify the normality of the data. Comparisons among groups were made by two-way ANOVA. One-way ANOVA was used for running distance, running time, running velocity, horizontal workload, and lactate variables. *Post hoc* combinations were performed using Bonferroni’s or LSD multiple comparison tests. Non-parametric results were compared using the Kruskal Wallis test. To find the effect size, Cohen’s *d*-test (Nakagawa and Cuthill, 2007) was applied by adopting the 0.02 to 0.15 points for a small effect, 0.16 to 0.35 medium effect, and greater than 0.35 as a large effect. The software SPSS (version 20.0) was used, considering *P* ≤ 0.05 as a significant value.

## Results

### Body and Cerebellum Weights

**FIGURE 2:**
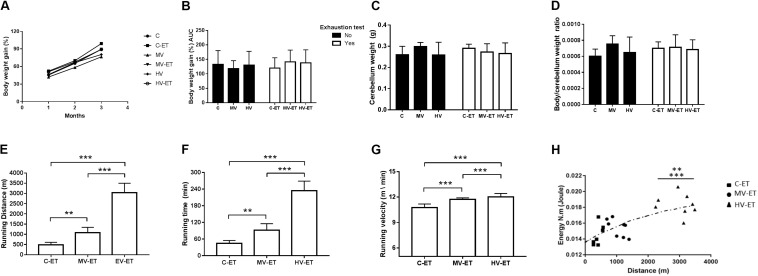
Somatic parameters and Running performance. **(A)** Body weight gain during months of training. **(B)** Area under the curve of body weight gain. **(C)** Cerebellum weight; **(D)** Body weight/cerebellum weight ratio. **(E)** Running distance of exhaustion test: MV-ET vs. C-ET; *P* = 0.002. HV-ET vs. MV-ET; C-ET; *P* < 0.001; **(F)** Running time of exhaustion test: MV-ET vs. C-ET; *P* = 0.002. HV-ET vs. MV-ET; C-ET; *P* < 0.001. **(G)** Velocity of exhaustion test HV-ET vs. MV-ET; C-ET; *P* < 0.001. **(H)** Horizontal Workload vs. running time during exhaustion test HV-ET vs. MV-ET (*P* = 0.002); C-ET; *P* < 0.001. C-ET, control + exhaustion test group; MV, moderate-volume group; MV-ET, moderate-volume + exhaustion test group; HV, high-volume group; HV-ET, high-volume + exhaustion test group. **P < 0.01; ***P < 0.001. Values represent mean ± S.D.

Figure 2A illustrates the percentage of body weight gain during the 3 months of training. No intergroup difference in the body weight was detected at the beginning of training [*F*(5,44) = 1.99, *P* = 0.101] and no difference in the percentage of body weight gain was achieved after 3 months of this training (Figure 2A), [*F*(5,43) = 0.908, *P* = 0.486]. The area under the curve of the percentage of body weight gain [*F*(5,44) = 0.188, *P* = 0.965] can be seen in Figure 2B. There was no difference in the cerebellar weight [Figure 2C; *F*(5,44) = 1.053 *P* = 0.401] or in cerebellum/body weight ratio among the groups [Figure 2D; (*F*(5,44) = 1.791, *P* = 0.137].

### Maximum Velocity Test

Table 2 describes the maximum velocity achieved by the animal groups during the 3 months of training. The training under moderate- or high-volume was able to progressively increase the velocity achieved by the animals when compared to the control condition [*F*(17,131) = 2.335, *P* = 0.004]. A significant increase was found between Vmax 2 vs. Vmax1 in MV; HV and HV-ET (*P* < 0.026). Vmax3 was also higher than Vmax1 in HV-ET and HV (*P* < 0.010). Intergroup differences were detected for Vmax 2 between HV vs. C (*P* = 0.021) and for Vmax3, increase was detected in HV-ET compared to C-ET (*P* = 0.016) and HV vs. C (*P* = 0.011).

**TABLE 2:** Maximum velocity test.

Groups	n	Vmax 1 (m/min)	Vmax 2 (m/min)	Vmax 3 (m/min)
C	8	23.8 ± 2.3	23.1 ± 2.5	21.3 ± 2.3
C-ET	7	20.8 ± 3.8	22.5 ± 5.2	21.4 ± 2.4
MV	8	20.8 ± 3.8	25.0 ± 4.0*	22.9 ± 2.7
MV-ET	7	23.1 ± 3.7	24.3 ± 3.2	24.4 ± 4.2
HV	8	22.9 ± 2.7	27.1 ± 2.6*‡	25.7 ± 1.9‡
HV-ET	7	21.3 ± 3.5	25.6 ± 4.1*	25.6 ± 3.2§#

Vmax, maximum velocity test. C, control group; C-ET, control group + exhaustion test; MV, moderate volume group; MV-ET, moderate volume group + exhaustion test; HV, high group; HV-ET, high group + exhaustion test. *P* ≤ 0.05; intragroup (* Vmax 2 vs. Vmax1, §Vmax 3 vs. Vmax1); intergroup (‡ HV or MV vs. C, # HV-ET or MV-ET vs. C-ET).

### Traveled Distance, Duration, Running Speed, and Horizontal Workload During Exhaustion Test

As expected, moderate- and high-volume training promoted a better running performance than the control condition during the exhaustion test (Figures 2E–H), evaluated by the running distance [*F*(2,20) = 129.019, *P* < 0.001], running duration [*F*(2,20) = 115.743, *P* < 0.001], running velocity [*F*(2,20) = 116.869, *P* < 0.001], and horizontal workload [*F*(2,20) = 15.748, *P* < 0.001]. The HV-ET group achieved greater running distance at the end of this test when compared to MV-ET (↑ 280.92%; *P* < 0.001; Cohen’s *d* = 5.13) and C- ET [↑ 624.75% (*P* < 0.001; Cohen’s *d* = 7.43]. The running distance traveled by MV-ET was also greater than that achieved by the C-ET group (↑ 222.39%; *P* = 0.002; Cohen’s *d* = 2.89) (Figure 2E). Similarly, the running duration (Figure 2F) in HV-ET animals was higher than in MV-ET (↑ 253.78%; *P* < 0.001 Cohen’s *d* = 4.79) and C-ET groups (↑ 522.00%; *P* < 0.001; Cohen’s *d* = 9.47. Longer running duration was achieved by MV-ET compared to C-ET (↑ 205.68%; *P* = 0.002; Cohen’s *d* = 5.62). The running speed (Figure 2G) and the horizontal workload (Figure 2H) in the MV-ET and HV-ET groups were also proportionally increased according to the training volume, when compared to the control condition (↑ 6.16 and ↑ 23,96%; *P* < 0.001; Cohen’s *d* = 0.74 and 2.60, respectively).

### Lactate, CK, Lactate Dehydrogenase, and Corticosterone Levels in the Serum

**FIGURE 3:**
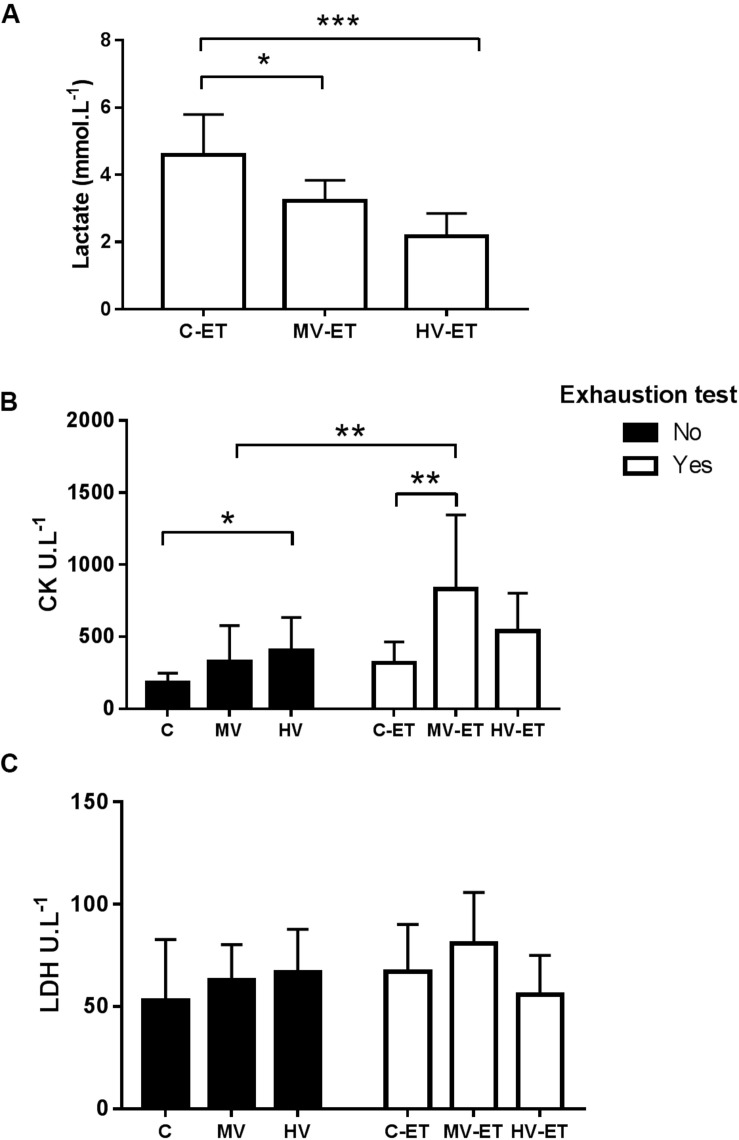
Serum levels of Lactate, Creatine Kinase and Lactate dehydrogenase. **(A)** Lactate: MV-ET vs. C-ET (*P* = 0.015); HV-ET vs. C-ET (*P* < 0.001). **(B)** CK: Creatine Kinase; C-ET (*P* = 0.026) and MV (*P* = 0.033) vs. MV-ET. HV vs. C (*P* = 0.015). **(C)** LDH: Lactate dehydrogenase. C, control group; C-ET, control + exhaustion test group; MV, moderate-volume group; MV-ET, moderate-volume + exhaustion test group; HV, high-volume group; HV-ET, high-volume + exhaustion test group. *P ≤ 0.05; **P < 0.01; ***P < 0.001. Values represent mean ± S.D. All the experiments were carried out in triplicate.

The ET provoked significant differences in the serum lactate concentration among the groups *F*(2,20) = 15.188, *P* < 0.001). Lower lactate levels were detected in the MV-ET (↓ 30.28%; *P* = 0.015; Cohen’s *d* = 4.69) and HV-ET (↓ 53.85%; *P* < 0.001; Cohen’s *d* = 2.48) groups when compared to C-TE (Figure 3A). Increased serum CK concentration was induced by the training or ET compared to the control condition [*F*(5,34) = 4.87, *P* = 0.002]. The MV-ET group presented higher values when compared to C-ET (↑ 161.68% *P* = 0.026; Cohen’s *d* = 1.34) and MV (160.72% *P* = 0.033; Cohen’s s *d* = 1.24) groups. In the HV group, CK values were greater than in the C animals (↑ 223.63%; *P* = 0.03; Cohen’s *d* = 1.31). No additional increase in the CK values was induced by the exhaustion test in the C-ET or HV-ET groups compared to their respective controls (C and HV) (Figure 3B). No statistical difference in serum LDH concentrations was detected among the groups [*F*(5,34) = 1.127, *P* = 0.368] (Figure 3C).

**FIGURE 4:**
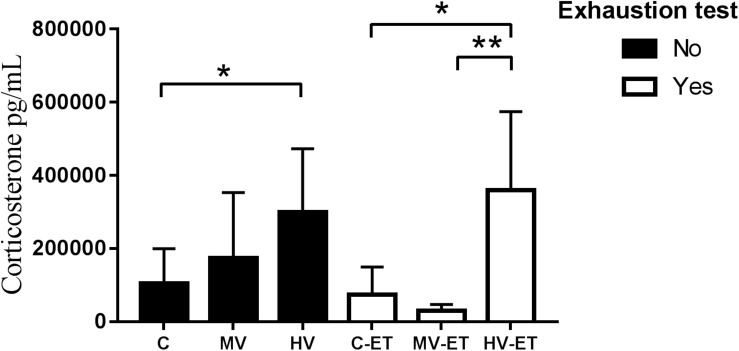
Corticosterone levels in the serum. HV vs. C (*P* = 0.038). HV-ET vs. MV-ET (*P* = 0.002) and C-ET (*P* = 0.013). C, control group; C-ET, control + exhaustion test group; MV, moderate-volume group; MV-ET, moderate-volume + exhaustion test group; HV, high-volume group; HV-ET, high-volume + exhaustion test group. *P ≤ 0.05; **P < 0.01. Values represent mean ± S.D. All the experiments were carried out in triplicate.

Figure 4 shows the results of serum corticosterone levels. A significant difference was found among the groups [*F*(5,27) = 4.008, *P* = 0.010]. The high-volume training *per se* induced higher levels of corticosterone compared to the control condition (↑ 44.44%; *P* = 0.038; Cohen’s *d* = 0.24). Values in HV were also greater than in MV-ET group (↑ 90.06%; *P* = 0.003; Cohen’s *d* = 0.01) and C-ET (↑ 75.17%; *P* = 0.022; Cohen’s *d* = 1.33). No intergroup difference was detected between the HV and HV-ET groups, but corticosterone values in the HV-ET group were elevated compared to MV-ET (↑ 91.74%; *P* = 0.002; Cohen’s *d* = 0.32) and C-ET (↑ 79.36%; *P* = 0.013; Cohen’s *d* = 0.68).

### Lipid Peroxidation in the Cerebellum

**FIGURE 5:**
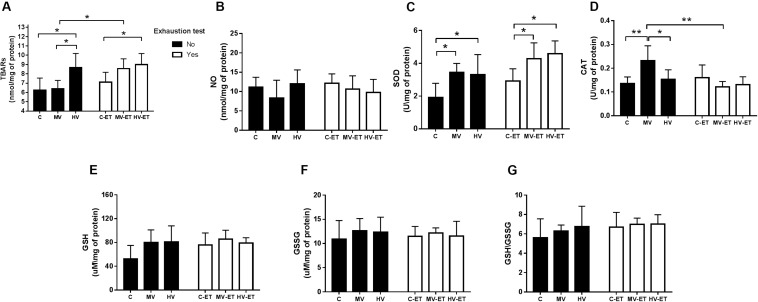
Oxidative Markers in the cerebellum tissue. **(A)** TBARS: MV-ET vs. MV (*P* = 0.021). HV-ET vs. C-ET (*P* = 0.032). HV vs. C (*P* = 0.036) and MV (*P* = 0.027). **(B)** NO: Nitric Oxide; **(C)** SOD: Superoxide dismutase; MV vs. C (*P* = 0.020); MV-ET and C-ET (*P* = 0.034); HV vs. C (0.048); HV-ET vs. C-ET (*P* = 0.011). **(D)** CAT: Catalase; MV vs. MV-ET (*P* = 0.001), HV (*P* = 0.043) and C (*P* = 0.006). **(E)** GSH: Glutathione; **(F)** GSSG: Oxidized Glutathione; **(G)** GSH/GSSG ratio. C, control group; C-ET, control + exhaustion test group; MV, moderate-volume group; MV-ET, moderate-volume + exhaustion test group; HV, high-volume group; HV-ET, high-volume + exhaustion test group. *P ≤ 0.05; **P < 0.01. Values represent mean ± S.D. All the experiments were carried out in triplicate.

Lipid peroxidation levels were differentially affected by the training or exhaustion test [*F*(5,27) = 3.396, *P* = 0.020]. Figure 5A illustrates increased levels of lipid peroxidation in the cerebellum of HV versus MV (↑ 26.1%; *P* = 0.027, Cohen’s *d* = 1.14) and C (↑ 27.9%; *P* = 0.036, Cohen’s *d* = 1.26); MV-ET vs. MV (↑ 25.2%; *P* = 0.021; Cohen’s *d* = 1.19). No difference was found between C-ET and C or between HV and HV-ET groups. Levels of malondialdehyde were also higher in HV-ET group vs. C-ET (↑ 20.9%; *P* = 0.032; Cohen’s *d* = 1.12) but did not differ when compared to values obtained in MV-ET.

### Total SOD and CAT Enzymatic Activity

Significant difference among the groups were detected for total SOD activity [*F*(5,27) = 5.295, *P* = 0.003]. High-volume training did not modify SOD activity when compared to moderate-volume training. Nevertheless, both volumes of training, increased SOD activity compared to the control condition: 44.02%; *P* = 0.01; Cohen’s *d* = 0.16 for MV vs. C and ↑ 41.81%; *P* = 0.02; Cohen’s *d* = 0.11 for HV vs. C groups. Similarly, MV-ET and HV-ET groups do not differ from each other but presented higher SOD activities when compared to C-ET (↑ 31.61%; *P* = 0.034; Cohen’s *d* = 0.15) (↑ 63.75%; *P* = 0.011; Cohen’s *d* = 0.21), respectively (Figure 5C).

Difference for CAT activity was also detected in the comparison among the groups [*F*(5,32) = 3.265, *P* = 0.02]. Higher activity was found in the MV group compared to MV-ET (↑47.82%; *P* = 0.001; Cohen’s *d* = 0.23), HV (↑34.78%; *P* = 0.043; Cohen’s *d* = 0.14), and C (↑41.44%; *P* = 0.006; Cohen’s *d* = 0.19) groups (Figure 5D). The exhaustion test did not increase CAT activity in none of the groups.

### Nitric Oxide (NO), Glutathione (GSH) and Oxidized Glutathione (GSSG) Levels

No intergroup difference was found for NO [*F*(5,35) = 1.123, *P* = 0.370] (Figure 5B), GSH [*F*(5,28) = 2.108, *P* = 0.103] (Figure 5E), or GSSG [*F*(5,28) = 0.265, *P* = 0.928] (Figure 5F) levels nor for GSH\GSSG ratio [*F*(5,28) = 0.775, *P* = 0.577] (Figure 5G) in the cerebellum.

### Interleukin-1β Levels

**FIGURE 6:**
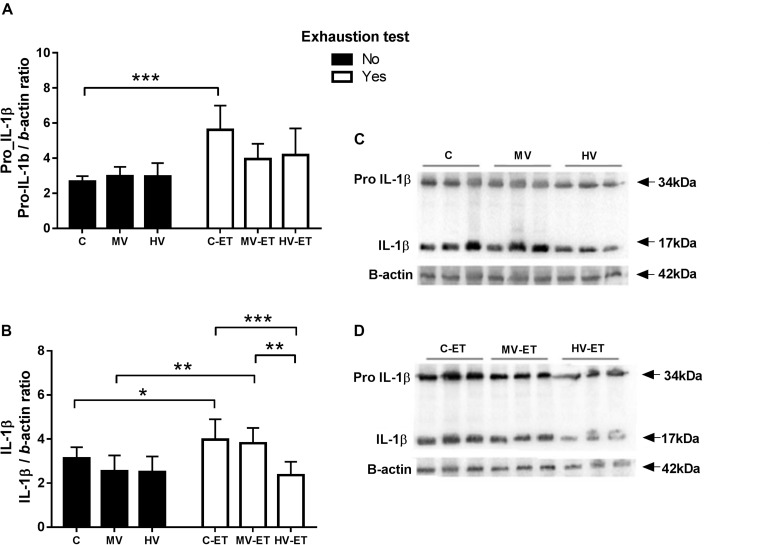
Pro- IL-1B and active IL-1b protein levels assessed by Western blot in the cerebellar tissue. **(A)** Pro IL-1B: C-ET vs. C (*P* < 0.001). **(B)** IL-1B: C-ET vs. C (*P* = c0.046); HV-ET (*P* < c0.001). MV-ET vs. MV (*P* = 0.006); HV-ET (*P* = 0.001). **(C)** Representative Western blotting membrane of groups without exhaustion test. **(D)** Representative Western blotting membrane of groups with exhaustion test. C, control group; C-ET, control + exhaustion test group; MV, moderate-volume group; MV-ET, moderate-volume + exhaustion test group; HV, high-volume group; HV-ET, high-volume + exhaustion test group. *P ≤ 0.05; **P < 0.01; ***P < 0.001. Values represent mean ± S.D. *N* = 3 independent experiments per group. All the experiments were carried out in triplicate.

A differential response among the groups was detected in the serum levels of pro-IL-1β (33–34 kDa) [*F*(5,35) = 7.396, *P* < 0.0001] and the active form of IL-1β (17 kDa) in the cerebellum [*F*(5,35) = 5.828, *P* = 0.001]. These levels were not modified by the training volume in both MV and HV groups when compared to the control condition. Nevertheless, 24 h after the ET, an increased concentration of pro-IL-1β was detected in the CT-ET compared to the C (↑53.57%; *P* < 0.001; Cohen’s *d* = 2.94). The amount of the active 17 kDa IL-1β was also intensified by the ET in the C-ET (↑22.92%; *P* = 0.046; Cohen’s *d* = 1.13) and MV-ET (↑33.15%; *P* = 0.006; Cohen’s *d* = 1.79) groups, while no change was detected in the cerebellum of HV-ET animals (Figure 6).

### GFAP Protein Expression Profile

**FIGURE 7:**
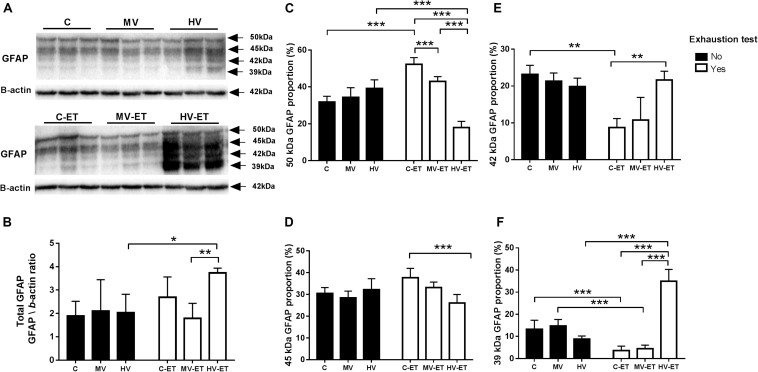
GFAP protein levels assessed by Western blot in the cerebellar tissue. **(A)** Representative Western blotting membrane of groups without and with exhaustion test. **(B)** Quantification of total GFAP (sum of the four isoforms): HV-ET vs. MV-ET (*P* = 0.004); HV (*P* = 0.023). **(C–F)**: The expression profile of each GFAP isoform. **(C)** 50 kDa GFAP: HV-ET vs. C-ET; MV-ET; HV (*P* < 0.001). C-ET vs. C (*P* < 0.001) and MV-ET (*P* = 0.013). **(D)** 45 KDa GFAP: C-ET vs. HV-ET (*P* < 0.001) **(E)** 42 kDa: C-ET vs. C (*P* = 0.001); HV-ET (*P* = 0.001). **(F)** 39 kDa GFAP: HV-ET vs. HV;C-ET;MV-ET (*P* < 0.001); C vs. C-ET (*P* < 0.001). MV vs. MV-ET (*P* < 0.001) C, control; C-ET, control + exhaustion test group; MV, moderate-volume group; MV-ET, moderate-volume + exhaustion test group; HV, high-volume group; HV-ET, high-volume + exhaustion test group. *P ≤ 0.05; **P < 0.01; ***P < 0.001. Values represent mean ± S.D. N = 3 independent experiments per group. All the experiments were carried out in triplicate.

Four GFAP isoforms were detected in the cerebellum using the Pan-GFAP antibody: the canonical GFAP protein with 50 kDa and the other three proteins with 45, 42, and 39 kDa. Figure 7 shows GFAP protein levels illustrated in two different ways. First, we analyzed the total amount of all GFAP isoforms among the groups [*F*(5,30) = 3.814, *P* = 0.011] and observed that the HV-ET group had higher values when compared to MV-ET (↑ 53.80%; *P* = 0.002; Cohen’s *d* = 4.31) but not to C-ET. This increase in the total GFAP in the HV-ET was also found when compared to the groups not submitted to exhaustion test: HV (↑ 46 71%; *P* = 0.015; Cohen’s *d* = 3.12) and MV (↑ 44.88%; *P* = 0.024; Cohen’s *d* = 1.79) groups.

In the separate analysis by isoform, no intergroup difference among C, MV, and HV groups was found for any of the four isoforms. On the other hand, the 50 kDa isoform increased in the C-ET and MV-ET when compared to C (↑ 36.52%; *P* < 0.001; Cohen’s *d* = 4.23 and ↑ 20.94%; *P* = 0.043; Cohen’s *d* = 2.30, respectively), MV (↑ 34.26%; *P* < 0.001; Cohen’s *d* = 3.94), and HV (↑ 36.50%; *P* < 0.001; Cohen’s *d* = 3.11). MV-ET and C-ET also differ (19.70%; *P* < 0.001; Cohen’s *d* = 3.73). On the other hand, in the HV-ET group, reduced levels of 50 kDa isoform were detected when compared to the other groups submitted to the exhaustion text: C-ET (↓ 65.56%; *P* < 0.001; Cohen’s *d* = 9.80), MV-ET (↓ 42.88%; *P* < 0.001; Cohen’s *d* = 9.44).

Regarding 45 kDa GFAP isoform [*F*(5,35) = 7.018, *P* < 0.0001] there was an increase in the C-ET when compared to HV-ET (↑ 30.76%; *P* = 0.004; Cohen’s *d* = 2) groups. The levels of 42 kDa GFAP did not differ among C, MV, and HV groups. However, in the C-ET, the values were lower (↓ 61.33%; *P* = 0.001; Cohen’s *d* = 6.07) than those found in the C (*P* = 0.001; Cohen’s *d* = 12.48), MV (*P* = 0.001; Cohen’s *d* = 5.30); HV (*P* = 0.004; Cohen’s *d* = 4.66) and HV-ET (*P* = 0.001; Cohen’s *d* = 5.31) groups.

The exhaustion test had a significant impact on 39 kDa GFAP isoform [*F*(5,32) = 51.56, *P* < 0.0001]. Although these levels did not change in the groups C, MV, and HV, they were reduced in C-ET group compared to C (↓ 27.08%; *P* < 0.001; Cohen’s *d* = 2.99) and in the MV-ET vs. MV (↓30.18%; *P* < 0.001; Cohen’s *d* = 4.35) groups. On the other hand, 39 kDa GFAP was abundant in the HV-ET group compared to HV (↑74.76%; *P* < 0.001; Cohen’s *d* = 6.75); MV-ET (↑87.29%; *P* < 0.001; Cohen’s *d* = 7,80), and C-ET (↓89.75%; *P* < 0.001; Cohen’s *d* = 7.82). These values were also elevated compared to the other group not submitted to the exhaustion test: MV (↓57.87%; *P* < 0.001; Cohen’s *d* = 0.47) groups.

## Discussion

The findings of the present study indicate that both the moderate- and high-volume training of aerobic exercise on a treadmill were able to reduce the rat body weight similarly and modify the running performance during ET simulating UE. Different responses of serum metabolic markers such as CK and corticosterone levels, as well as in cerebellum oxidative status, were observed in the animals trained under the two distinct conditions of exercise volume. Three months of high-volume training at moderate intensity impaired the cerebellum antioxidant defense system, resulting in lipid peroxidation. Moreover, when high-volume training was associated with ET, an altered GFAP isoform profile was detected in this brain region.

### Running Performance and Serum Lactate Levels

To ascertain whether our exercise training volume did indeed enhance muscular function and provoke adaptive responses, we performed exercise-exhaustion tests on treadmills simulating UE. Although there is no consensus on the definition of UE in rodents, events completed at 4 (Millet et al., 2011) or 6 h of running (Turner et al., 2014) are considered comparable to UE in humans. Our results showed that trained animals submitted to the ET maintained considerable aerobic resistance for up to 4 h. In the animal groups submitted to moderate- or high- training volume, a lower serum lactate concentration was detected 24 h after ET compared to the control. In the control condition, serum lactate concentration exceeded the anaerobic threshold, which typically can be characterized by a lower mitochondrial oxidative capacity, phosphocreatine depletion, increased H^+^, and recurrent substrates for adenine nucleotide catabolism (Sahlin et al., 1990; Lewis et al., 2010; Marcinek et al., 2010). The lower lactate concentration observed in the trained groups agreed with previous studies on rodents, that long-term training under aerobic conditions can induce this response, associated to physiological and muscular adaptations such as high mitochondrial sensitivity in oxidizing the pyruvate and muscle angiogenesis and a greater gluconeogenesis capacity (Gaesser and Poole, 1988; Voltarelli et al., 2002; Brito Vieira et al., 2014; Booth et al., 2015; Abreu et al., 2016). Modifications in the liver metabolism accompanied by an increase in the liver monocarboxylate transporter 2 protein levels have also been found in mice submitted to moderate-intensity treadmill exercise for 6 weeks (Lezi et al., 2013).

In long-distance competitions in humans, muscle metabolic changes are accompanied by lactate elevation (Lewis et al., 2010) mostly due to the maximum effort exerted during competition. In our study, different from this, there was no intensity variation during ET. Furthermore, the UE simulation was performed at a lower intensity (50% of maximum speed) than during the preceding protocol of training (70%). Therefore, the findings observed in the MV and HV groups are in agreement with previous evidence that rats submitted to running training under aerobic threshold present lactic stability (Abreu et al., 2016). Moreover, it has been well established that aerobic endurance exercise increases fat consumption as an energy source by decreasing glucose oxidation and pyruvate production (Ferreira et al., 2016).

### Creatine Kinase Levels

Ultra-endurance exercises also promote a catabolic state by activating intracellular proteolytic enzyme activity, which can promote muscle protein degradation and augmented cell permeability, allowing the leaking of cell contents into the circulation (Dohm et al., 1987; Baird et al., 2012). For this reason, plasma CK is often used as a biomarker for myocardial and skeletal muscle damage or changes in myocyte membrane permeability after exercise (Yamashita and Yoshioka, 1991). An increase of plasma CK has been also observed in rats after running, even in the absence of histological damage (Kuipers, 1994). In the present study, a significant rise in the serum CK levels was detected in the HV group, and this increase was maintained without modification 24 h after ET. On the other hand, the insult induced by ET was able to elevate CK levels in the MV group. Two peaks in the serum CK levels have been reported after a marathon run and intense exercise, associated with early transient changes in the membrane permeability and a late inflammatory response during 24–48 h of recovery (Clarkson and Ebbeling, 1988). On the other hand, other studies in humans have shown that plasma CK concentration after ultramarathon runs (∼200 Km) declines to basal levels after 24 h of recovery (Son et al., 2015).

The serum CK concentrations here detected in the MV-ET and HV groups were higher than those observed after moderate-intensity protocols in animal models (De Araujo et al., 2012) even when submitted to the eccentric contraction exercise by downhill running (Isanejad et al., 2015) or even when performed at a high intensity (Choi et al., 2013; Rezaei et al., 2017). Wistar rats, when subjected to progressively intense exercise, can reach anaerobic thresholds at velocities exceeding 15 m/min and a maximum CK concentration close to 350 U/L at 17.5 m/min (Rezaei et al., 2017). In the MV-ET and HV groups, higher serum CK concentrations were detected under the aerobic threshold, at a velocity not exceeding 12 m/min and in the absence of increased levels of LDH in the serum. Hepatic and renal function can also become impaired after UE (Lipman et al., 2014; Jastrzȩbski et al., 2015; Knechtle and Nikolaidis, 2018). Acute kidney injury is relatively common in ultramarathon races (Hoffman et al., 2013; Lipman et al., 2014) and post-race serum CK and creatinine concentrations have been significantly correlated in humans (Hoffman and Weiss, 2016; Hodgson et al., 2017). Exhaustive swimming exercise has been shown to provoke kidney injury in rats (Wu et al., 2012). Although we did not investigate the renal function and histological markers in the muscle of MV-ET and HV animals, we cannot discard the possibility that their increased serum CK levels were related to different types of muscle or renal insults inducing modifications in the clearance of CK (Hodgson et al., 2017). It is possible that that the moderate intensity of the aerobic exercise adopted in the present study could be at least partially responsible for the lack of increased serum LDH levels. Adopting the correlation reported by Rodrigues et al. (2007) between VO2 max and the maximum velocity achieved by the animals in our study, we estimated that the predicted VO2 max was on average 70.5 ± 1.7(mL/Kg/min -1) between 10.78 and 12.9 m/min. Thus, our findings in rodents are in agreement with previous evidence that when aerobic exercise is performed at a value inferior to 80% VO2 max, the release of CK 24 h after the running in the treadmill is not accompanied by increased serum LDH levels, indicating the absence of cardiac stress (Callegari et al., 2017). On the other hand, recent evidence from studies in humans demonstrated that an ultra-endurance mountain race performed under low intensity and aerobic conditions simultaneously increased CK and LDH levels in the athletes (Ramos-Campo et al., 2016).

### Serum Corticosterone Levels

Serum corticosterone levels were also elevated by the training volume in the HV animals when compared to the control or MV groups. However, similarly to what was detected for CK, no additive effect was induced by the ET. Various aspects must be considered in a discussion of these findings. It is well established that exercise induces increased glucocorticoid secretion by stimulation of the hypothalamus-pituitary-adrenal (HPA) axis (Chennaoui et al., 2002). Karkoulias et al. (2008) demonstrated that marathon running in humans causes plasma cortisol elevation just after running and the recovery to basal levels may take a week. According to these authors, the degree of cortisol elevation is dependent on the duration of the run. The importance of glucocorticoids in determining muscle strength and endurance lies in their catabolic effects, especially hyperglycemic effects, and facilitates the conversion of proteins to glycogen, as well as providing amino acids for gluconeogenesis (Chen et al., 2017). These effects are probably involved in the relative resistance of the HV groups. Bernardi et al. (2013) also reported increased corticosterone levels concomitant to reduced serum lactate concentration in rats submitted to treadmill exercise for 4 weeks (20 min/day, 5 days/week during 4 weeks). In addition to acting on the glucose metabolism, increased serum corticosteroid levels may participate in the maintenance of homeostasis and the development of physical fitness as an adaptive mechanism in trained subjects (Mastorakos and Pavlatou, 2005). In the HV group, recovery of the corticosterone to basal values did not occur 24 h after the training period, and the exhaustion test did not modify these levels in the HV-ET animals. It has also been shown that under the long-term exercise of moderate intensity, adaptive mechanisms reducing the sensitivity to glucocorticoids in target tissues including HPA axis can occur both in rats (Chennaoui et al., 2002) and in human marathon runners and triathletes (Duclos et al., 2003, 2007; Duclos and Tabarin, 2016). These mechanisms can prevent deleterious effects of corticosterone (in rodents) or cortisol (in humans) elevation, decreasing additional muscle insult. It remains to be investigated if this type of adaptive mechanism occurred in the HV-ET group.

### Effects on the Antioxidant Resource of the Cerebellum

The main hypothesis of the present study was that rodent cerebellar resilience to oxidative injuries could be reduced after high training volumes, regardless of the simulation of UE running. Indeed, we found LP in the HV, MV-ET, and HV-ET groups but not in the MV group. These data indicated that the LP detected was especially due to unbalanced levels of enzymatic antioxidant resources. An intensified CAT activity occurred only in the MV group, concomitant with an increased SOD activity, which was able to efficiently degrade the hydrogen peroxide (H_2_O_2_) produced by this latter enzyme.

Usually, the exhaustion test on the treadmill can induce systemic oxidative stress depending on the intensity or muscle fatigue (Acikgoz et al., 2006). In the MV-ET group, most of the animals displayed a relative resistance, reaching their point of exhaustion after 2 h of running. The unbalanced levels of the SOD/catalase ratio in the cerebellum associated to the increased levels of systemic CK detected in this group indicated that the previous moderate-volume training for 3 months was not able to confer resistance to a UE simulation, neither by the peripheral organs nor by neural regions involved in locomotion control. Chalimoniuk et al. (2015b) reported increased levels of LP in the cerebellum of rats after 6 weeks of moderate exercise performed during 60 min/day. Nevertheless, no lipid damage was detected in the cerebral cortex of the same animals where balanced levels of SOD and CAT were found (Chalimoniuk et al., 2015b). Falone et al. (2012) also reported increased antioxidant enzyme activity in the cerebral cortex after 16 weeks of training (5 days/week for only 20 min). Despite the differential vulnerability of these brain regions to oxidative insults, the present data reinforce the hypothesis that high-volume training *per se* can be deleterious to the cerebellum even under moderate intensity. However, it is not clear at this moment why the exhaustion test did not provoke an additive effect on the HV group.

Unchanged levels of NO production were here detected in the trained animals either under moderate or high volume when compared to the control condition. Under physiological conditions, NO has been implicated in cerebellar long-term depression and long-term potentiation, which are forms of synaptic plasticity involved in motor learning (Ogasawara et al., 2007). NO also plays an important role in matching blood flow to oxygen demand in the brain during exercise (Toda et al., 2009). A 6-week exercise training protocol on the treadmill (5 days/week), beginning with 40 min at 16 m/min and gradually increased to 60 min/day at 28 m/min was able to enhance nitrergic transmission in the cerebellum, striatum, and midbrain but not in the cortex and hippocampus of adolescent and adult rats (Chalimoniuk et al., 2015a, b). On the other hand, a comparative study analyzing the effect of different modalities of exercise reported different proportions of nitrergic activity in the hippocampus, striatum, and cerebellum (Torres et al., 2006). According to this latter study, only acrobatic exercises increased NADPH-diaphorase activity in the cerebellar granular layer when compared to sedentary and other exercise modalities including the use of a treadmill for 30 min a day, at 12 m/s during 33 days.

The lack of changes in the NO levels inside the cerebellum of MV, MV-ET, HV, and HV-ET groups in the present study was accompanied by an unmodified GSH/GSSG ratio. Crosstalk between NO and GSH has been described as an important mechanism necessary for modulating the cell redox status (Baldelli et al., 2019). Within the cells, besides its antioxidant effect, GSH also has the function of buffering the flux of NO. It has been demonstrated that reduced levels of GSH can trigger a severe NO imbalance, causing neuronal death (Aquilano et al., 2011a). Even a slight and non-toxic decrease of GSH in the brain caused protein nitration which was reversed by inhibiting NO production (Aquilano et al., 2011b). Some studies have analyzed the impact of the exercise of moderate or elevated intensity. These have reported increased levels of GSH in the brain. Nevertheless, in a systematic review of the effects of aerobic training on redox status, Camiletti-Moirón et al. (2013) did not find a homogeneous response of GSH, GSSG, and GPx, but a slight tendency to a positive effect of aerobic exercise on these parameters. In a recent meta-analysis, we also did not find any tendency for decreased or increased GPx activity after moderate or elevated exercise intensity or volume among several brain regions (de Souza et al., 2018). Therefore, similar values of GSH/GSSG ratio and NO levels between control and trained groups detected in the present study suggest that even after high-volume training and ET a physiological response can still be maintained for keeping the redox balance, favoring modulatory effects of NO in the cerebellum motor function. Future studies are necessary to address this subject.

#### Inflammatory Markers and Astrocyte Reactivity Profile

Long-term exercise at low-to-moderate intensity has been documented as an important modulator of neuroinflammation and glial activation, inducing adaptive responses (Mee-Inta et al., 2019). The gene expression of IL-1β can be brought about by several transcriptional elements such as cAMP-responsive element, NF-κB, among others (Shirakawa et al., 1993) that can be activated by hypoxia, oxidative stress, hyperosmolarity, thermal injury, gamma radiation, and microbial stimuli (Haddad and Harb, 2005). Translation depends on the activation of MAP kinases producing pro-IL-1β, which must be cleaved by the cysteine protease caspase-1 to adopt biological activity as IL-1β (Hewett et al., 2012). In the healthy brain, the levels of the pro-IL-1β and the active IL-1β are low, and this interleukin can stimulate a variety of signaling pathways, including those involved in synaptic plasticity (Hewett et al., 2012). On the other hand, IL-1β levels in the brain can be increased by local damage and/or peripheral inflammation (Pitossi et al., 1997; Laye et al., 2000; Hewett et al., 2012).

In the present study, moderate and high training volumes did not affect the pro-IL-1β or the active 17-kDa IL-1β levels in the cerebellum. Thus, the lipid peroxidation detected in the group HV was neither correlated with increased levels of IL-1b nor associated with ET. Only in the MV-ET group higher levels of IL-1b occur concomitant to increased lipid peroxidation levels. On the other hand, in the CT-ET group, the amount of Pro-IL-1b and the active IL-1b was elevated by the ET even in the absence of lipid peroxidation. These findings suggest a higher sensitivity of the cerebellum of untrained rats to transcriptional or translational effects on IL-1b levels when submitted to UE simulation. It has been shown that fatigue elicited by the exhaustive test may involve disturbances in the immune system, which can be hampered by long-term training (Chennaoui et al., 2002; Petersen and Pedersen, 2005; Morgado et al., 2012). Our findings indicated that only in the HV-ET animals was the previous training able to prevent the activation of IL-1b induced by the ET, but the mechanism involved in the finding is not clear.

Brain IL-1β is mainly produced by microglia, peri-vascular infiltrating macrophages (Herx et al., 2000), and astrocytes (Rappold and Tieu, 2010) but can be released from some neurons (Silverman et al., 2005) under pathological conditions. In the cerebellum, for example, Purkinje neurons in neonates undergo apoptosis and excitotoxic death when IL-1β and TNF-α levels are released by microglia after acute hypoxia (Kaur et al., 2014). However, current literature also reports that low-intensity exercise is sufficient to reduce the release of IL-1β in part due to modifications in the microglia phenotype (Mee-Inta et al., 2019).

Furthermore, an imbalance in antioxidant and anti-inflammatory neuroprotective mechanisms may also be related to astrocyte reactivity (Liddell, 2017). The relationship between running, pro-inflammatory cytokines, and glial cell activation have been studied in some models of brain injury (Ang et al., 2004). This fact motivated an analysis of the GFAP expression profile in the cerebellum considering the importance of Bergman glia for the homeostasis of this region, locomotor performance, and antioxidant resource (Fernandez-Fernandez et al., 2012). Astrocytes also play trophic, metabolic, and neuronal support functions (Matsui et al., 2017). Besides, they are actively involved in glutamate and GABA metabolism, which contribute to ATP synthesis (Magi et al., 2013). These beneficial actions of astrocytes characterize their neuroprotector phenotype, also called A2 (Cunningham et al., 2019). On the other hand, when overactivated, astrocytes can be neurotoxic acquiring an A1 phenotype and are the main targets of IL-1β released by microglia (Liddell, 2017). Under this condition, astrocytes have a reciprocal interaction with IL-1β, leading to the production of reactive oxygen species (Rappold and Tieu, 2010), and IL-1β can modify the physiological state of Bergman cells.

Exercise can induce astroglial proliferation depending on the brain’s demand for energy (Li et al., 2005). The GFAP gene can be alternatively spliced giving rise to at least nine proteins that differ from the first described isoform, called GFAPα with 50 kDa: GFAPβ, GFAPγ, GFAPδ/ϵ, GFAPk, GFAPΔ135, GFAPΔ164, GFAPΔexon6, GFAPΔexon7, and GFAPζ (review in Middeldorp and Hol, 2011). Usually, astrocyte reactivity is accompanied by increased levels of GFAPα protein isoform, which is phosphorylated and mainly expressed in mature and proliferating stages. Augmented levels of this GFAP isoform have also been related to inflammatory status under some pathological conditions (Catts et al., 2014; Sullivan, 2014).

Analyzing the GFAP protein expression, we identified four distinct isoforms in the cerebellum with 50, 45, 42, and 39 kDa, respectively. We have found that neither moderate- nor high-volume training modified the cerebellum GFAP expression profile of these isoforms when compared to the control condition. However, the exhaustion test provoked in the C-ET and MV-ET increased levels of the 50 kDa GFAP isoform, while it concomitantly reduced the 42 and 39 kDa isoforms. Higher levels of the 50 kDa GFAP isoform found in the C-ET were also simultaneous with a higher amount of Pro-IL-1b and the active form of IL-1b. In the HV-ET group, an opposite effect was found in the GFAP isoform profile, where the 50 kDa isoform was about 40% of that found in the control animals and 50–60% of the values in the cerebellum of C-ET, MV-ET, and HV groups. On the other hand, augmented levels of 42 and 39 kDa GFAP were found in HV-ET.

Changes in the expression level of the GFAP isoforms influence the intermediate filament network, which can modify astrocyte mobility and structure (Sullivan, 2014; Sereika et al., 2018). Usually, GFAP phosphorylation contributes to the extensive remodeling of glial networks in mitosis and can also affect its interactions with other intracellular proteins (Moeton et al., 2014). In some neurodegenerative diseases as well as in the astrocytoma of grade IV, GFAP isoforms with lower molecular weight were better expressed when compared to the control condition (Hol et al., 2003; Sereika et al., 2018). Reactive gliosis associated with a drop in GFAP phosphorylation has been reported, for example, in the neurodegenerative condition in Parkinson’s disease (Clairembault et al., 2014). It is not clear at this moment which mechanisms are involved in the changed GFAP isoform profile detected here in the cerebellum of the HV-ET group. A recent study using proton magnetic resonance spectroscopy demonstrated that acute exhaustive endurance exercise in rats previously trained on a treadmill increased glutamate signals in the cerebellum. These findings suggested astrocyte dysfunction causing disequilibrium in the glutamate-glutamine cycle and a delay in the return of glutamine from these glial cells to neurons (Swiątkiewicz et al., 2017). Therefore, the modified GFAP profile found in the cerebellum of the HV-ET group suggests impaired astrocyte reactivity. Nevertheless, it remains to be investigated if this molecular change contributes to the lack of response to oxidative and inflammatory insult induced by the UE simulation.

## Conclusion

In conclusion, the results corroborate the initial hypothesis, indicating that rat cerebellar resilience to oxidative damage is maintained during 3 months of moderate-volume training, but high-volume training at moderate intensity impaired the enzymatic antioxidant defense system of this brain region. Also, we demonstrated for the first time that UE simulation after high-volume training can alter the GFAP isoform profile, suggesting impaired astrocyte reactivity in the cerebellum. The findings also indicate that moderate-volume training, under the aerobic condition for 3 months, does not confer resistance to UE simulation in rats, either for systemic markers or the oxidative and inflammatory status of the cerebellum. Altogether, the data highlights the importance of further studies in other brain regions involved in the movement control given the increase in the number of participants in ultra-marathons nowadays.

## Data Availability Statement

The datasets generated for this study are available on request to the corresponding author.

## Ethics Statement

The animal study was reviewed and approved by Animal Use Ethics Committee (CEUA) of the Federal University of Pernambuco, Brazil (# 0035-2017).

## Author Contributions

RS, BA-C, and SM: designing the experiment. FS, RS, and DP: running training. DP, RA, RS, LG, FS, and GM: biochemical and western blot analysis and data curation. RS and BA-C: writing – original draft. All authors contributed to the final version of the manuscript.

## Conflict of Interest

The authors declare that the research was conducted in the absence of any commercial or financial relationships that could be construed as a potential conflict of interest.
